# Improving Ghana’s mental healthcare through task-shifting- psychiatrists and health policy directors perceptions about government’s commitment and the role of community mental health workers

**DOI:** 10.1186/s12992-016-0199-z

**Published:** 2016-10-01

**Authors:** Vincent Israel Opoku Agyapong, Conor Farren, Eilish McAuliffe

**Affiliations:** 1 Department of Psychiatry, Faculty of Medicine and Dentistry, University of Alberta, 1E1 WMC 8440 112 St NW, Edmonton, AB T6G 2B7 Canada; 2 Department of Behavioural Sciences, Kwame Nkrumah University of Science and Technology, Kumasi, Ghana; 3 Centre for Global Health, University of Dublin, Trinity College, Dublin, Ireland; 4 Department of Psychiatry, University of Dublin, Trinity College, Dublin, Ireland; 5 School of Nursing, Midwifery and Health Systems, University College Dublin, Dublin, Ireland

**Keywords:** Task-shifting, Psychiatrists, Community mental health workers, Health policy directors

## Abstract

**Background:**

The scarcity of mental health professionals places specialist psychiatric care out of the reach of most people in low and middle income countries. There is growing interest in the effectiveness of task shifting as a strategy for targeting expanding health care demands in settings with shortages of qualified health personnel.

Given this background, the aim of our study was to examine the perceptions of psychiatrists and health policy directors about the policy to expand mental health care delivery in Ghana through a system of task-shifting from psychiatrists to community mental health workers (CMHWs).

**Methods:**

A self-administered semi-structured questionnaire was developed and administered to 11 psychiatrists and 29 health policy directors. Key informant interviews were also held with five psychiatrists and four health policy directors. Quantitative data were analysed using descriptive statistics. Qualitative data were analysed thematically.

**Results:**

Almost all the psychiatrists and 23 (79.3 %) health policy directors were aware of the policy of the Government of Ghana to improve on the human resource base within mental health through a system of task-shifting. Overall, about half of the psychiatrists and 9 (31 %) health policy directors perceived there is some professional resistance to the implementation of the policy of task shifting. The majority of respondents were of the view that CMHWs should be allowed to assess, diagnose and treat most of the common mental disorders. The respondents identified that CMHWs usually perform two sets of roles, namely; officially assigned roles for which they have the requisite training and assumed roles for which they usually do not have the requisite training. The stakeholders identified multiple challenges associated with current task shifting arrangements within Ghana’s mental health delivery system, including inadequate training and supervision, poor awareness of the scope of their expertise on the part of the CMHWs.

**Conclusion:**

Psychiatrists and health policy directors support the policy to expand mental health service coverage in Ghana through a system of task-shifting, despite their awareness of resistance from some professionals. It is important that the Government of Ghana upholds its commitment of expanding mental healthcare by maintaining and prioritizing its policy on task shifting and also providing the necessary resources to ensure its success.

## Background

One of the cornerstones of primary health care reforms advocated by the World Health Organisation in the 2008 World Health Report is achieving universal health coverage and reducing health inequalities [1]. However, the severe shortage and imbalanced distribution of a trained health workforce poses a serious threat to achieving universal health coverage, including mental health at the required scale [2]. Mental disorders constitute 14 % of the overall burden of disease and 28 % of the non-communicable disease burden [3]. They also account for a third of the years lived with disability by adults globally, and are five of the ten leading causes of disability [4]. However, across the entire economic spectrum—and especially in low- and middle income countries—public sector resource allocation for mental health is disproportionately low [5], and this from a pie that is already much too small [6]. For example mental health care in Ghana, which is the focus of this research, is government funded; receiving only 0.5 % of the overall health budget, or about 0.007 % of GDP [7, 8]. The scarcity of mental health professionals, places specialist psychiatric care out of the reach of most people in low and middle income countries (LAMICs) [9, 10], particularly in the lowest income countries and in rural/low-income regions within countries [11]. More so than other areas of medicine, mental health care relies on trained workers, rather than technology or tools [5] which means a shortage of trained mental health professionals results in significantly greater unmet mental health needs among populations. In recent years, there is growing interest in the effectiveness of task shifting as a strategy for targeting expanding health care demands in settings with shortages of qualified health personnel [12]. Some authors have proposed to alternative term ‘task sharing’ to the term ‘task shifting’ as the later seem to be more in tune with the idea of rationally redistributing tasks among health workforce teams in other to make efficient use of available human resources for health [13, 14]. In 2011 for example, Ghana had only 11 psychiatrists in active service providing for the mental health needs of a population of nearly 25 million people and almost all of these psychiatrists were based in the southern half of the country [15]. Ghana has therefore adopted a system of task-shifting to address the critical shortage of psychiatrists needed to meet the health needs of its over 25 million inhabitants [16–19]. The related policy document of Ghana’s Ministry of Health describes the origins and the rational for the task-shifting arrangements within Ghana’s mental health delivery system [20]. The bulk of modern mental health care at the community level in Ghana is therefore currently provided by Community Mental Health Officers (CMHOs), Clinical Psychiatric Officers (CPOs) and Clinical Psychiatric Nurses (CPNs). These health cadres together are generally described as Community Mental Health Workers (CMHWs) in Ghana. The training and job descriptions as well as how long these health cadres have been operating in Ghana have been described in detail in related publications [16–18]. In summary, CPNs AND CMHOs are responsible primarily for case detection in the community, referral of patients to CPOs and Psychiatrists and for follow-up of patients within the community who are on medication to monitor adherence with medication and attendance of appointments. CPOs on the other hand are responsible for diagnosing and treating a range of common psychiatric conditions. It has been suggested that implementation of task shifting by legally transferring the tasks of physician-clinicians to non-physician-clinicians or other cadres may not only be counterproductive to the quality of health services, but may also undermine the professional distinction of those who have spent many years to earn such professions [21]. There can therefore be resistance by higher cadres given perceived flattening of hierarchal structures [22], and the additional supervisory responsibilities that more skilled staff must assume [23, 24]. There is thus the suggestion that there are some institutional and professional resistances to the introduction of task shifting models of care [25]. Given this backdrop, our study aims to examine the perception of psychiatrists and health policy directors about the government’s policy of expanding mental health care delivery in Ghana through a system of task shifting and the role community mental health workers play as part of these task shifting arrangements. The research questions included:

1. Do psychiatrists and health policy directors in Ghana acknowledge that task shifting exists within the mental health delivery system and is the Government of Ghana committed to expanding mental health services care through task shifting?
2. What do psychiatrists and health policy directors perceive should be the scope of practice of community mental health workers in Ghana’s mental health services as part of task-shifting arrangements? What are the challenges related to scope of practice for these cadres?
3. What value do psychiatrists and health policy directors place on task-shifting arrangements within Ghana’s mental health delivery system?

## Methods

The study design was a mixed-methods study with a cross-sectional survey and qualitative interviews. The study setting has been described in related publications [16, 17]. For the quantitative study, total sampling methods were used, where by all psychiatrists nationally, and all health policy directors from the Eastern Region of Ghana who were attending an annual performance review meeting in the regional capital Koforidua were targeted for inclusion in the survey. A self-administered semi-structured questionnaire with optional answers including Likert scales and open-ended questions was developed by the research team based on a review of literature on similar studies of task shifting using community health workers. The questionnaire was pretested and revised based on the results of the pre-test before being administered to the respondents. They generally took 15 min to complete and no monetary or other incentives were provided to the respondents. Sections of the questionnaire assessed the perceptions of the psychiatrists and health policy directors about the existence of task shifting within Ghana’s mental health delivery system, government’s commitment to expanding on mental health care through a system of task shifting and the role and scope of practice of the CMHWs which are the focus of this paper. For the qualitative part of the study, in-depth interviews were conducted with five psychiatrists and four health policy directors selected from three regions of Ghana. Every other psychiatrist approached and requested to participate in the quantitative study was also invited to participate in the key informant interviews. For the health policy directors, two of those attending an annual performance review meeting in Koforidua were randomly selected and invited to participate in the key informant interview. In addition, two health policy directors from two other regions of Ghana were approached and interviewed.

All interviews were conducted in English and tape recorded. In addition, responses to open ended questions on the survey forms were analysed together with the interview data.

The study received prior institutional review board approval from the Health Policy and Management and Global Health Ethics Committee at Trinity College Dublin (Reference number: 07/2013/06) and the School of Medical Sciences, Kwame Nkrumah University of Science and Technology, Kumasi, Ghana (Reference number: CHRPE/AP/300/13). The study also received the approval of the office of the Chief Psychiatrists of the Ghana Health Service. All study participants received information leaflets about the study and provided written consent prior to completing the questionnaires.

Data were collected between 10th of August 2013 and 30th of October 2013. Quantitative data from categorical variables including Likert scales were analysed using descriptive statistics with SPSS version 20. For the qualitative data, all interview transcripts and open-ended survey responses were analysed using thematic analysis which is the most common form of analysis in qualitative research [26]. First, structural coding was used to generate initial codes in line with the specific research questions. Pattern coding, which allows identification of explanatory or inferential codes was further applied to the initial codes to identify patterns or emerging themes and subthemes across the dataset. This second cycle coding used the deductive approach of thematic analysis in that generation of the initial themes and subthemes were directed by each of the four overall research questions of the study. The candidate themes and subthemes were further reviewed by checking their “fit” with the collated extracts for each theme and subtheme as well as with the dataset overall. The final sets of themes and subthemes relevant to the overall research questions/objectives of the study are reported and supported with verbatim quotes.

## Results

For the quantitative study, all 11 psychiatrists in active service in four of the ten regions who were approached (100 % response rate for psychiatrists) and 29 out of 33 regional and district health policy directors working in 27 districts of Ghana agreed to participate in the survey (87.87 % response rate for health policy directors). For the qualitative study, key informant interviews were held with 5 psychiatrists and two health policy directors.

### Results of the quantitative study

#### Perception of health policy directors and psychiatrists about task shifting within Ghana’s mental health delivery system

In all, 26 (89.7 %) of the health policy directors believed that the human resource base within Ghana’s mental health delivery system was either completely inadequate (3 (10.3 %)) or inadequate (23 (79.3 %)). In comparison, all the psychiatrists believed that the human resource base in Ghana is completely inadequate.

Ten of the psychiatrists reported that they were aware that task shifting exists within Ghana’s mental health system. Only one psychiatrist reported that he was not aware of task shifting within Ghana’s mental health delivery system. Similarly, only 3 (10.3 %) health policy directors believed that task-shifting does not exist within Ghana’s mental health delivery system.

When asked if they are aware of a policy of the Government of Ghana to improve on the human resource base within the mental health delivery system through task shifting, almost all the psychiatrists responded that they were aware of such a policy, with only one psychiatrist responding that he was not aware of such a policy. Nine out of these ten psychiatrists said they believed the government is committed to implementing the policy of task shifting to improve on Ghana’s mental health delivery. In comparison, 23 (79.3 %) health policy directors expressed that they were aware of a policy of the Government of Ghana to increase the human resource base within the mental health delivery system through task-shifting and these respondents all expressed an opinion that the Government of Ghana is committed to implementing such a policy. The rest (6 or 20.7 %) expressed no awareness of a policy of the Government of Ghana to increase the human resource base within the mental health delivery system through task shifting.

About half of the psychiatrists said they believed there is some resistance to the implementation of task shifting within Ghana’s mental health delivery system. They identified resistance from the three groups of professionals including traditional and spiritual healers, some psychiatrists and some Community Psychiatric Nurses. In comparison, 9 (31 %) health policy directors perceived there are resistances from certain quarters towards implementing a policy of task shifting within Ghana’s mental health delivery system including resistance from psychiatrists and psychologists.

#### Scope of work for the CMHWs -quantitative data

All the psychiatrists said they worked closely with CPNs, 10 (90.1 %) said they worked closely with CPOs while only 6 (54.5 %) said they worked closely with CMHOs. In comparison, 13 (44.8 %) health policy directors said they work closely with CMHOs, 11 (37.9 %) said they work with CPOs/MAPs while 22 (75.9 %) said they work closely with CPNs.

**Fig. 1:**
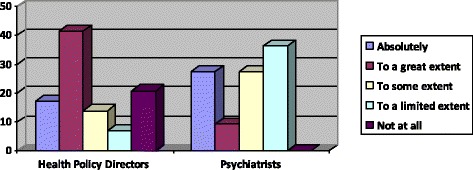
Percentages of the extent to which psychiatrists and health policy coordinators perceive that CMHWs should be allowed to independently assess patients

**Fig. 2:**
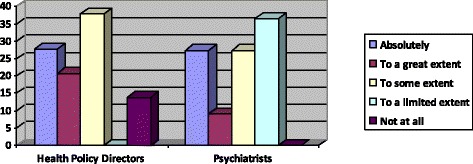
Percentages of the extent to which psychiatrists and health policy coordinators perceive that CMHWs should be allowed to independently diagnose mental health conditions

All the psychiatrists agreed that CMHWs should independently assess and diagnose patients with mental health conditions although to varying extents as shown in Figs. 1 and 2.

In comparison, 23 (79.3 %) health policy directors expressed the view that all community mental health workers should be allowed to assess patients independently while 25 (86.2 %) said they should be able to diagnose mental conditions independently also to varying extents as shown in Figs. 1 and 2.

Table 1 gives the number (percentage) of psychiatrists and health policy directors who expressed the view that CMHWs should be able to diagnose or treat specific mental health conditions respectively.

**Table 1:** Number (Percentage) of psychiatrists and health policy directors who expressed the view that community mental health workers should be able to diagnose or treat specific mental health conditions respectively

Mental health condition	CMHWs should be able to diagnose independently *N* (%)		CMHWs should be able to treat independently *N* (%)	
	Psychiatrists	Health Policy Directors	Psychiatrists	Health Policy Directors
Schizophrenia	10 (90.9 %)	25 (86.2 %)	9 (81.8 %)	25 (86.2 %)
Mood disorders	10 (90.9 %)	25 (86.2 %)	10 (90.9 %)	25 (86.2 %)
Anxiety disorders	8 (72.7 %)	23 (79.3 %)	9 (81.8 %)	25 (86.2 %)
Eating disorders	5 (45.5 %)	19 (65.5 %)	2 (18.2 %)	18 (62.1 %)
Personality disorders	5 (45.5 %)	22 (75.9 %)	1 (9.1 %)	18 (62.1 %)
Addiction disorders	11 (100 %)	23 (79.3 %)	5 (45.5 %)	14 (48.3 %)
Child mental health conditions	6 (54.5 %)	14 (48.3 %)	2 (18.2 %)	5 (17.2 %)
Other mental health conditions	2 (18.2 %)	2 (6.9 %)	3 (27.3 %)	0 (0 %)

All the psychiatrists and health policy directors were of the opinion that all CMHWs should be able to offer counselling, prescribe medication and visit patients in their homes as part of their job description.

In all, 5 (45 %) psychiatrists said they believe many patients will not have obtained any mental health care if the CMHWs were not in their current posts.

In comparison, 14 (48.3 %) health policy directors believed the mental health patients living in their districts will not obtain any mental health care if the CMHWs currently working there were not in their posts. Overall, 3 (27.3 %) of the psychiatrists said there are no clear policy guidelines regulating the work of CMHWs, 4 (36.4 %) said there are some policy guidelines while the remaining 4 (36.4 %) said there are limited policy guidelines. Similarly, 5 (45.5 %) of the psychiatrists said there are no clear treatment protocols to guide the work of CMHWs with the remaining 6 (54.5 %) reporting that there are limited treatment protocols in place to guide the work of CMHWs.

On the other hand, 3 (10.3 %) of the health policy directors said there are no clear policy guidelines to regulate the work of the CMHWs and 10 (34.5 %) said there are no clear treatment protocols to guide the work of the CMHWs.

### Results of the qualitative analysis

#### Scope of work for the CMHWs

The following themes and subthemes were identified in relation to psychiatrists and health policy directors’ perceptions about the role and scope of practice of CMHWs in Ghana: range of roles (officially assigned roles and assumed roles), task shifting value, and task shifting challenges.

**Range of roles**: This theme describes participants’ perceptions about the range of roles carried out by CMHWs. Two subthemes emerged as officially assigned roles and assumed roles.

***Officially assigned roles***: Participants were of the view that for all CMHWs, their officially assigned roles include identifying, assessing, referring, and following-up on mental health cases; and engaging in public education. The roles of diagnosing, prescribing medication, and counselling were reported by some participants to include the officially assigned roles of CPOs and CPNs. Some participants reported that CPOs and CPNs were also officially expected to be able to treat or manage, at least to a limited extent, conditions including anxiety disorders, depression, and schizophrenia.

*“They [CPOs] function broadly like the physician…..i.e. the management of common psychiatric disorders and they are supposed to have been trained to recognize those conditions that they need to send on to the psychiatrist.”* Psychiatrist 3.

Regarding CPNs however, some were of the view that their officially assigned duties exclude diagnosing and treating mental health cases. *“I was shocked at the conference when the coordinator of CPN told us that they are backed by law to diagnose and treat. I got up and said no! You are a nurse you go into the community to identify new cases for onward referral to the psychiatrist for diagnosis and treatment then you follow up in the community to ensure they are compliant”* Psychiatrist 3.

Participants indicated that CMHOs were not supposed to engage in CPO– and CPN–designated duties such as diagnosing, prescribing medication, and treating mental health cases, or were only supposed to carry them out to a limited extent. However, they were expected to be able to carry out other assigned tasks such as visits to homes and community places (e.g. prayer camps and durbars), and ensuring medication adherence and relapse prevention.

*“The CMHOs they are not supposed to treat actually. [They are not supposed to treat at all?] At all”* Psychiatrist 5.

*“I think their (CMHOs) core function is support, is to find cases, bring them to the CPNs or CPOs and make sure that the patients in the communities are taking their medication and not relapsing.”* Psychiatrist 2.

#### Assumed roles

All participants admitted that CMHWs often assume the roles of their superiors. That is, CHMOs may assume the roles of CPNs, CPOs, and/or psychiatrists. Similarly, participants reported that CPNs and CPOs may also assume the roles of psychiatrists. In fact, some participants reported that there seems not to be a clear delineation between the roles actually played by CPOs, CPNs, and CMHOs.

*“The CMHOs are not supposed to prescribe and treat but as we are all aware currently they are doing so”* HPD 1.

*“We have duplication of roles. We have CMHOs behaving like the CP’s and CPNs behaving like CPOs and the CPOs behaving like psychiatrists. In some cases we have the CMHOs behaving like psychiatrists”* Psychiatrist 3.

### Task shifting value

This theme describes participants’ perceived value of CMHWs assuming some of the mental health roles of their superiors. Specifically, participants viewed the idea of task-shifting as:

#### Necessary and the way to go

Some respondents expressed the view that in light of the inadequate numbers of trained psychiatrists in Ghana, it is necessary to bridge the gap in mental health care by using CMHWs to provide the care traditionally provided by psychiatrists.

*“Well task shifting in the short term and maybe even in the medium term is necessarily what we should do because I have observed the mental health seed in Ghana for the last 40 years and there is hardly been a change in the ratio of the psychiatrist to the population…..the numbers being attracted to mental health are still not enough. Very low you know so for a long time to come I believe that we will need to use these schedule of people so task shifting will be required.”* Psychiatrist 4.

*‘Community mental health workers are an absolute need but they need adequate and proper supervision’.* HPD 2.

*‘Training of more community mental health workers is highly recommended as the training of psychiatrists is a lot more expensive and highly unattractive for young doctors so Ghana may not be able to bridge the treatment gap without them’.* HPD 3.

#### Helpful in reducing workload

Some participants expressed the view that task-shifting from psychiatrists to CMHWs is necessary to reduce the workload on the few psychiatrists working within Ghana’s mental health delivery system.

*“It’s’ a very good idea, because the psychiatrist alone cannot work in the mental health hospital and send the patient to the regions and continue to work all alone. You go to a big hospital and there is only one doctor. There is a limit to what you can do so they are quite happy for these nurses to help them”* HPD 2.

*“If anything at all it reduces it (workload). We were able to send patients to the community and we were confident that these people were being seen. So it reduces it.”* Psychiatrist 5.

One psychiatrist however expressed the need for more psychiatrists to be trained in tandem with the training of more CMHWs so that the psychiatrists could provide training and supervision for the CMHWs: *“To make it efficient we still need deliberate policies to increase the numbers of psychiatrists even if it is for the purpose of training these people because there are just too few psychiatrists to do the job as well as do the training which is essential, the supervision and all that.”.* Psychiatrist 5.

#### Useful in helping to meet mental health needs and addressing the problem of distance

Participants identified a benefit of task-shifting as its ability to meet the mental health needs of citizens locally rather than rely on the care provided by the few psychiatrists located miles away in urban cities.

*“They [psychiatrists] recognise the fact that they are few and they cannot be everywhere and those people [CHMWs] that others are seeing if those people were not there they will be left unseen.”* Psychiatrist 5.

*‘If more community mental health workers are trained and dispatched into the communities, it would improve on mental health care and reduce psychotic patients roaming in our streets’.* HPD 1.

*‘They help a lot in early mental health detection and reduce the number of cases admitted to the psychiatric hospitals’.* HPD 2.

#### Beneficial in promoting security and confidence among community members

Some respondents perceived that task shifting from psychiatrists to CMHWs gives the community the needed security and confidence that their mental health needs will be met.

*“Their presence in the community alone affords people the security that they need to deal with mental health cases in the community…..can give medication and contact in case the patient is relapsing and that gives the families confidence and then you can manage the patient.”* HPD 4.

#### Better than some alternative or no treatment

While some participants expressed concerns about the risks of task shifting such as the risk of misdiagnosis, some participants believed that assumption or task-shifting of roles, even if it may compromise on quality, is better than not providing any mental health care for community members at all.

*“It [actual roles performed by CMHWs] wouldn’t have been [performed if CMHWs were not at post]. It would have just been left to traditional healer to do whatever they want to do with the patient.”* Psychiatrist 3.

*“It’s better than prayer camps. We say better than nothing. I suspect it will be at worse neutral.”* Psychiatrist 2.

### Task shifting challenges

This theme describes participants’ perceptions about the factors that challenge the provision of quality mental healthcare through task shifting or assumption of roles by CMHWs. Inadequacy and recognition were the two subthemes that emerged.

#### Inadequacy (in training, supervision, and resources for assumed roles)

For psychiatrists and HPDs, whilst the current training for CMHWs may be adequate in relation to performing their officially assigned duties, it is inadequate in preparing them to effectively deliver the mental health roles that they often assume. Participants mentioned that the training for CMHWs is characterised by less practical work and a short duration, and it is also shallow in content (e.g. excludes prescribing and treatment for CMHOs).

*“It [training received by CMHWs] adequately prepares them for the role they are supposed to play but….Not the role they are currently playing. Not the role some of them are taking upon themselves”* Psychiatrist 5.

*“I do know that it’s (training for CMHOs) a very low level of training. It is one year of training and I don’t know what goes into it. But I can’t imagine much, and I can’t imagine how much of it is practical and how much of it is also psychological.”* Psychiatrist 2.

Participants further reported that CMHWs receive inadequate supervision for the roles they play within the communities due to factors such as scarcity of psychiatrists. Also, the support of other health workers available to CMHWs was reported to be inadequate.

*“I think that because our psychiatrists there are so few of them and they are so weighed down even their ability to supervise or to grow the CPNs and CPOs is limited.”* Psychiatrist 2.

*“there are just too few psychiatrists to do the job as well as do the training which is essential, the supervision and all that”* Psychiatrist 4.

*“We also have support for the community psychiatric nurses in terms of other workers in social care those are very few and therefore inadequate.”* HPD 2.

Additionally, participants mentioned that lack of resources (including medications and conditions of service) for mental health work, as well as lack of stakeholder interest in mental health were other factors that influence the quality of the actual mental health duties that CMHWs perform within the communities.

*“All that they have are medications which are erratic in supply…..Sometimes they can go for a year without supply. In the last three years the whole country has been on a drug holiday. Some of them don’t even have an office or a consultation area, a detention area for acute cases for observation. With the human resource we still have one CPN to a very large area or two to a whole district which really is a lot of work. Then they don’t have vehicles for moving around. They don’t have motor bikes or even bicycles that they will use for transport. They rely on the district assembly which most times are not forth coming.”* Psychiatrist 3.

***Recognition (recognition of role limitations by CMHWs)***: Psychiatrists expressed concern that CMHWs often take up responsibilities beyond their limitations and discussed that to effectively shift tasks within the mental health delivery system with a goal to increase access to quality mental healthcare, it is important that CMHWs recognize the limits of their roles.

*“I’ve had the opportunity to interact with my colleagues in other areas and they have huge challenges with the CPOs holding themselves out as qualified psychiatrists which is not so”* Psychiatrist 3.

*“What I think we need to do is to streamline it (training) more for them also to be able to appreciate their role and to stay within their limitations”* Psychiatrist 5.

*“Another thing they would need is for them to know exactly their limits, particularly CMHOs”* Psychiatrist 5.

***Lack of treatment protocols and guidelines***: Participants identified the lack of clear treatment protocols to guide the work of the CMHWs as a hindrance to the effective delivery of their work.

*“‘The community mental health workers do a great job which can’t be quantified so they should be well equipped with appropriate treatment protocols so they can continue to do their job even better’.* HPD 3.

*“Maybe there are protocols but I am not aware of this because I think that for the kind of training they have and for their level I think there should be no ambiguity about making diagnosis about treatment and that they should not feel that they should just be able to handle everything……every case. I am saying this because I don’t think I see or certainly I don’t get enough referral from them. Whilst I am happy they are able to handle a lot of cases I suspect there are quite a lot that they are not able to handle”.* Psychiatrist 2.

## Discussion

Our study examined the perception of psychiatrists and health policy directors about the government’s policy of expanding mental health care delivery in Ghana through a system of task shifting and the role community mental health workers play as part of these task shifting arrangements as detailed in a related policy document [20]. Overall, our study has established that all psychiatrists and almost all health policy directors in Ghana perceive the human resource base within Ghana’s mental health delivery system to be inadequate. The landmark *Lancet* global mental health series of 2007 and 2011, along with WHO’s ‘*Atlas: Mental Health Resources in the World’*, highlighted the major shortages of psychiatrists, psychiatric nurses, psychologists, and social workers in LAMICs [5, 27, 28]. In 2007, the World Health Organisation estimated that of the 21.6 million people living in Ghana, 650,000 were suffering from a severe mental disorder and a further 2,166,000 were suffering from a moderate to mild mental disorder [29]. With only 32,283 people receiving treatment in 2007, the WHO further estimated that the treatment gap was 98 % of the total population expected to have a mental disorder [29]. It is therefore re-assuring that our study found that almost all the psychiatrists and the majority of health policy directors believe that the Government of Ghana is committed to expanding mental healthcare through a system of task shifting and they were aware of the existence of a policy to this effect. It has also been suggested that any long term success of task shifting hinges on serious political and financial commitments and that task shifting requires careful attention to organization, structure and resourcing of health services [30]. Samb et al. also called on governments and international and bilateral agencies to help prepare health systems to successfully implement task shifting by ensuring the establishment of appropriate regulatory frameworks and the building of training and management capacity [31]. In Uganda, one study reports that there are no legal instruments, written policy or guidelines to support sector-wide task shifting, existing practice was permissive of task shifting within obstetric care, with no strict enforcement of “scope of practice” rules. Thus task shifting was happening often without legal protection for those who took on delegated tasks within the existing regulatory framework, save for institutional arrangements that permitted such task shifting [32]. CMHWs in Ghana may therefore have better legal protection compared to their counterparts engaging in task shifting within obstetric care in Uganda. Furthermore, in Tanzania, concerns have been raised about how health personnel policy issues related to how new tasks from higher cadres or other cadres or skilled professionals to lower/other/unskilled cadres could be aligned with their job descriptions and what implications this has for their promotion and career development [21].

Stakeholders in our study ascribed multiple reasons why task shifting is practiced or necessary to advance Ghana. One of the key themes that emerged from stakeholders was that task shifting is better than some alternative or no treatment as exemplified by the quote from one of the psychiatrists: *“it’s better than prayer camps. We say better than nothing. I suspect it will be at worse neutral.” Psychiatrist 2.* When tasks have been shifted from traditional professional cadres (e.g. specialists, doctors or nurses) to new professional cadres, most studies compare the new cadre’s productivity and patient outcomes to the traditional cadre’s. The parallel comparison occurs between higher- and lower-skilled workers. However, the appropriate comparison is between the results from the care received by the new cadre and the results from the care the patient would have received–if any care at all–had the new cadre not been available [33]. Verteuil articulated this point well in his response to Kruk et al.’s Mozambique study: “An appropriate comparator to tecnicos de cirurgia would be a ‘do nothing’ comparator as opposed to using formally trained surgeons....a more realistic alternative for patients treated by tecnicos de cirurgia would be no formal treatment at all, which would, it is presumed, result in far worse outcomes for the patients” [34]. Therefore arguably, there is some rational for using CMHWs to augment mental healthcare delivery in Ghana, as the alternative will be no conventional mental healthcare for the majority of the population. The theme of “better than nothing” closely relates to another theme of “useful in helping to meet mental health needs and addressing the problem of distance” identified by other stakeholders and exemplified by this quote from another psychiatrist: *“they [psychiatrists] recognise the fact that they are few and they cannot be everywhere and those people [CHMWs] that others are seeing if those people were not there they will be left unseen.” Psychiatrist 5.* Task shifting is often introduced to enable professional workers to focus on more technical, life-saving roles and to expand coverage of effective interventions in areas with limited health personnel [12]. Another objective of task shifting is to reduce the time needed to scale up the health workforce, because the cadres performing the shifted tasks require less training [33]. A related theme which emerged was that task shifting helps to reduce the workload. While task shifting does not increase the number of qualified staff, delegating roles can mitigate a health system’s dependence on highly skilled individuals for specific services [35]. Thus, the primary objective of task shifting is to increase productive efficiency. That is, to increase the number of health care services provided at a given quality and cost, or, alternatively, to provide the same level of health care services at a given quality at a lower cost [33].

A final theme that emerged relates to security and confidence among community members as illustrated by this quote from the regional director of health services: “their presence in the community alone affords people the security that they need to deal with mental health cases in the community”-Regional Director of Health Services. Task shifting is welcomed for its potential to bring about more efficient use of health personnel while diverging from efforts that have previously failed, such as government post assignments or extensive medical training [36, 37]. Rather, the approach emphasizes inclusion and, in some cases, development of a lower level cadre to assume tasks originally intended for higher level cadres. Increased retention of health workers, especially in remote areas, is another reason for implementing task shifting strategies. In a Tanzanian study, informants had a consistent perception that it is easier to maintain a lower cadre health worker who is already working in the remote health facility than deploying qualified personnel from somewhere else, particularly in urban areas [21].

This study reveals that more than half of psychiatrists perceive that there are only limited treatment protocols or guidelines to assist CMHWs in managing mental health cases in the community. In comparison, about a third of all health policy directors were not aware of the existence of any treatment guidelines for the CMHWs. The success of task shifting depends on many factors including, but not limited to, proper supervision [38] and the existence of proven indicators (tests or criteria) that can be used by health care workers without the benefit of much experience, to make good management decisions. The latter requirement, that there be reliable indicators, is critical in any scheme to shift tasks from highly competent specialist medical personnel with in-depth knowledge to generalist health workers with limited specialist knowledge, experience, and equipment, while avoiding an unacceptable decrease in quality of care. The development of successful algorithms and training for primary health care workers is based on likelihoods that specific, easily identifiable signs (indicators) reflect certain pathophysiologic processes [39]. For example in mental health, several validated instruments exist to guide clinicians with diagnosis and these could be incorporated into an assessment kit for the CMHWs to guide them in categorising mental health conditions. The incorporation of such clinical tools or indicators will make it possible to reasonably expect a minimally experienced generalist cadre to manage specialist medical conditions. If such indicators cannot be identified then it may not be reasonable to expect “task shifting” to be successful. Useful indicators need to be simple to use and should have high sensitivity and specificity [39]. The lack of consensus among all our study participants regarding the existence of clear treatment protocols need to be examined more closely and addressed at the policy level. Clear and simplified diagnostic and treatment guidelines need to be incorporated in the training of CMHWs and made available to them before they are posted to their communities. For a start, more information and/or training should be made available to educate the existing workforce about task-shifting initiatives for mental health in Ghana. Furthermore, for task shifting to be successful, legal protections and liabilities, and the regulatory framework for task shifting should be designed to accommodate new task shifted practices [40]. In this regard, even though the CMHW posts were all established by the Ministry of Health, a third of all psychiatrists and about one in ten health policy directors reported they were not aware of the existence of clear policy guidelines which regulate the work of the CMHWs.

Consistent with reports in sections of the literature [23, 24], our study has established that about half of all psychiatrists and a third of all health policy directors perceive that there is some resistance to the policy of expanding on mental health care delivery in Ghana using a system of task shifting. They identify resistance from psychiatrists, psychologists, CPNs and traditional healers. However, in an international survey conducted by the World Psychiatric Association on ways to reduce the treatment gap for mental disorders, psychiatrist respondents acknowledged the important role of non-specialist providers (primary care doctors, nonmedical health workers) in diagnosis, medication management and psychosocial support, suggesting that the psychiatrists are in support of task shifting within mental health delivery systems [41]. Consistent with this international survey, and notwithstanding the perceived resistance to the implementation of task shifting from psychiatrists and other health cadres, our study finds there are considerable variation in the levels of support amongst the psychiatrists and health policy directors surveyed in Ghana, for CMHWs to be allowed to independently assess and diagnose mental health conditions. Significantly, the majority of psychiatrists and health policy directors are in support of CMHWs treating common mental health conditions including schizophrenia, mood disorders and anxiety disorders.

Whilst our study did not directly measure the effectiveness of task shifting from psychiatrists to CMHWs, a number of themes emerged as factors which hinder the use of task shifting to expand mental health delivery in Ghana. One important theme related to the inadequacy of the training of CMHWs for the role they actually play within the mental health delivery system. It seems important for there to be ongoing training for these CMHWs. The importance of ongoing training has been highlighted in the literature. Community health workers in South Africa [42] report a desire for better training and supervision to meet the formidable challenges posed by the synergy of HIV, tuberculosis and poverty. As discussed above, some CMHWs are compelled by the absence of other health workers in their districts to perform functions for which they have no training, functions which would otherwise have been performed by other higher level cadres if they were at their post within the district. This theme also relates to CMHWs who assume higher level roles without formal training even when there are other higher level cadres to perform those roles. One study conducted in Zambia found that additional training needs were identified by almost 85 % of lay counsellors [43].

Stakeholders also identified inadequate resources as one of the factors hindering CMHWs from performing their roles, including the erratic supply of medicines, lack of office accommodation and lack of transportation to undertake community visits. With regards to solutions, stakeholders suggested that the training of CMHWs be streamlined and that they are clearly informed what their roles are and to understand that they have limitations. It is important for mental health policy makers to stress to lesser trained health cadres the importance of recognising their limitations, and to seek to refer patients’ in situations where the patient’s mental or physical health issues are beyond what they can manage based on their training and experience.

Stakeholders also stressed the importance of government providing adequate resources including constant supply of medication and transportation to enable the CMHWs to function effectively. The literature suggests that the successful integration of new health cadres into health care delivery requires the availability of reliable medium- to long-term funding, with time frames of 20 to 30 years instead of 3 to 5 years [44]. Not only remuneration, but funding for training, supervision and infrastructure support must be ensured [30]. It will therefore be necessary for the Government of Ghana to ring-fence the medium to long term funding for mental healthcare so that CMHWs and other mental health workers in Ghana can more effectively discharge their duties.

Our study has a few limitations which have been described in related publications [16]. First, although the survey questionnaires used for the quantitative part of the study were well researched, based on the study objectives and pre-tested and revised before use, they are nonetheless not validated instruments and the individual items may not have accurately measured what they set out to measure. Secondly, only 11 of the 18 psychiatrists working in Ghana could be contacted although repeated efforts were made to contact all of them. It is possible that those psychiatrists who were not contactable will have varied perspectives about task shifting within Ghana’s mental health delivery system. Similarly, for logistic and other reasons, the policy directors were from only 27 of the 216 districts of Ghana with the majority working in the Eastern Region, which means their views may not been nationally representative and this may limit the generalizability of our findings. In addition, an even smaller number of psychiatrists and health policy directors were included in the qualitative study. This notwithstanding, large sample sizes are not required for studies in which the researcher is attempting to attain a grounded understanding of a phenomenon, to generate working hypotheses which means the low sample size in our study does not diminish the relevance of our findings.

## Conclusions

This study highlights several important reasons for task shifting as well as gaps in the service provision at the community level within Ghana’s mental health delivery system. The study has also established that most psychiatrists and health policy directors in Ghana support the Government’s policy to expand on mental health care delivery through a system of task-shifting, despite their knowledge of resistance from certain professionals. Most psychiatrists and health policy directors in Ghana are also in favour of CMHWs assessing, diagnosing, and independently treating common mental health conditions although to varying extents. It is important that the Government of Ghana keeps up to its commitment of expanding mental healthcare by maintaining and prioritizing its policy on task shifting and also providing the necessary resources to ensure its success. Mental health policy makers and coordinators also need to thoroughly review the training curriculum, and also evaluate the job descriptions of all CMHWs in Ghana to ensure that they are consistent with the demands and healthcare needs of patients they care for in their communities. There is also the need for measures to be put in place to monitor and measure the effectiveness of task-shifting arrangements within Ghana’s mental health delivery systems, so that any lapses in services delivery can be more quickly identified and addressed.

## Abbreviations

CMHO: Community mental health officer
CMHW: Community mental health worker
CPN: Community psychiatric nurse
CPO: Clinical psychiatric officer
LAMICs: Low and middle income countries
WHO: World Health Organisation
